# Before the algorithm: An exemplar case of the necessity of statistical testing for epidemiological consistency in public health data

**DOI:** 10.3934/publichealth.2026008

**Published:** 2026-01-20

**Authors:** Marco Roccetti

**Affiliations:** Department of Computer Science and Engineering, University of Bologna, Bologna, Italy

**Keywords:** biostatistics, computational epidemiology, inferential statistics, public health data, selection bias

## Abstract

The adoption of sophisticated analytical tools, including Machine Learning and massive data processing, has accelerated health research. However, a foundational principle asserts that the rigor of these complex methods is dependent on the integrity and validity of the underlying statistical design. I posit that advanced analyses, particularly in epidemiology, must be subsequent to the rigorous verification of methodological coherence. In this study, I used an exploratory case to demonstrate a crucial cautionary principle: Complex models amplify, rather than correct, substantial methodological limitations. To demonstrate this, I applied standard descriptive and inferential statistical methods (Z-tests, Confidence Intervals, and t-tests) alongside established national epidemiological benchmarks to a published cohort study on vaccine outcomes and psychiatric events. Through this approach, I identified multiple, statistically significant inconsistencies within the source data, including implausible incidence rates and relevant baseline group imbalances. These findings, supported by inferential statistical evidence, demonstrated that the observed effects (e.g., contradictory Hazard Ratios) are not biological but are mathematical artifacts stemming from uncorrected selection and classification biases in the cohort construction. These paradoxes arise from the exclusion of prevalent psychiatric cases in the vaccinated group and the misclassification of pre-existing conditions as new incident events in the control group. Our analysis serves as a robust demonstration that the validity of any conclusion drawn from subsequent advanced ML or statistical modeling sourced from public health data rests on first passing the test of basic epidemiological consistency.

## 1. Introduction

The modern era of medical research is defined by an escalating reliance on Big Data platforms and Machine/Deep Learning (ML/DL) algorithms [1]. These technologies, ranging from neural networks for medical image analysis to predictive models for population health, promise to uncover subtle associations and forecast patient outcomes with high precision. The prevailing narrative often suggests that the complexity of the computational approach inherently guarantees the robustness and reliability of the conclusions. Instead, this creates a problematic methodological inversion where complex modeling precedes basic data validation [2],[3]. In fact, research demonstrates that simpler models, when properly specified, can yield identical or more stable results than overly complex computational methods [4],[5].

However, a core principle of data science remains immutable and should be the *sine qua non* for any complex analysis: The outcome of any processing, regardless of its computational sophistication, is ultimately constrained by the quality and design integrity of the input data. A flawed methodological foundation, particularly in medical cohort construction or case definition, will not be corrected by the power of complex statistics or ML/DL. Instead, the complexity may mask and amplify the underlying bias. This is why the application of ML/DL and Big Data tools in public health research must be rigorously conditioned on the initial validation of the cohort through descriptive and inferential statistical methods and the consistency of its observed epidemiological metrics.

For example, in retrospective observational studies utilizing large administrative databases (such as national health service cohorts), the crucial challenge lies in achieving a covariate balance between comparison groups. Failure to correct for intrinsic, large differences in baseline characteristics (like age, comorbidity status, and health-seeking behavior) introduces severe selection and misclassification bias. The resulting statistical metrics, such as Hazard Ratios (HRs), would then reflect this baseline disparity rather than any true biological effect.

In this paper, I leverage the power of basic descriptive and rigorous inferential statistics (e.g., Z-tests and t-tests) alongside established national epidemiological benchmarks to identify an uncorrected selection bias in a population-based study [6]. It is important to emphasize that our research is intended as a methodological illustration. I use the study reported in [6] as an exemplar case, aiming to demonstrate that foundational statistical scrutiny is a mandatory prerequisite that must be satisfied before any subsequent ML/DL or predictive analysis is meaningful. Our objective is to prove that simple methodological scrutiny is the definitive test for validity, highlighting that observed health outcomes may be epidemiologically inconsistent and indicating that reported associations are likely the results of flawed data input rather than biological signals in the absence of the aforementioned scrutiny. In essence, the failure to execute this foundational step may increase the likelihood that subsequent complex models, no matter how sophisticated, will propagate a systematic error, rendering it useless for public health decision-making. The remainder of the paper is structured as follows: The Materials and Methods section (Section 2) details our consistency check approach, including the use of inferential statistical tests. In Section 3, I present the three major epidemiological statistical inconsistencies observed in the scrutinized data, which were derived from checks rooted in basic statistical epidemiology. Had these simple consistency checks not been applied, the data could have proceeded to advanced modeling, inevitably producing an invalid model for subsequent public health inference. In Section 4, I discuss how advanced models like ML/DL would yield invalid or biased results on such flawed data, emphasizing the urgent necessity of basic yet robust covariate balancing techniques. Finally, Section 5 provides a summary conclusion of our finding.

## 2. Materials and methods

### 2.1. Sources of data

The data analyzed in this report are sourced from the literature, specifically from the reported incidence rates and baseline characteristics extracted directly from the primary study under examination [6]. In this study, I utilize administrative data to compare outcomes over a three-month follow-up period between a vaccinated group and an unvaccinated control group. Given the nature of the case study, where I investigate the association between COVID-19 vaccination and adverse psychiatric events, it is necessary to perform preliminary data validation and calculations to ensure the integrity of subsequent inferences. For this analysis, I focus, in detail, on key psychiatric outcomes, notably: 1) Schizophrenia (ICD-10: F20-F29), 2) Bipolar Disorder (ICD-10: F31), and 3) Anxiety Disorder (ICD-10: F41.x). Instead, the epidemiological benchmarks used for comparison are derived from robust, independent national studies focused on the South Korean population (subject of the investigated case), which provide validated annual prevalence and incidence rates for the psychiatric conditions studied [7]–[9]. It should be also acknowledged that national administrative data may exhibit slight variations compared to regional cohorts. However, given the large-scale nature of the Seoul population-based study [6], these national benchmarks provide the most authoritative reference for assessing the epidemiological plausibility of the observed rates.

### 2.2. Descriptive statistics

Here, I list and explain the descriptive statistics methods, and their corresponding formulas, used for basic epidemiological checks on the study data under scrutiny.

#### 2.2.1. Calculation of epidemiological consistency metrics

To assess the validity of the reported incidence rates and Hazard Ratios (HRs) of the scrutinized case, I applied standard statistical methods based on consistency checks and known relationships between epidemiological measures. To begin, all national annual incidence and prevalence rates were normalized to the equivalent three-month period and to the per 10,000 population scale used in the primary study [6] for direct comparison using the conversion formula for annual incidence *I(Annual)* to estimated quarterly incidence *I(Quarterly)*, expressed as:

I(Quarterly)=I(Annual)/4 (1)

#### 2.2.2. Calculation of expected upper bounds

For Anxiety Disorders particularly, the reported 12-month prevalence *P(Annual)* was used to establish an absolute theoretical upper limit for the incidence over three months [9]. Since in computational epidemiology the *incidence* (new cases) must be lower than its *prevalence* (total existing cases), the quarterly fraction of the national prevalence serves as the maximum plausible quarterly incidence *P(Quarterly_max)*, as shown below:

P(Quarterly_max)=P(Annual)/4 (2)

#### 2.2.3. Consistency checks on HRs

The HR is the ratio of the hazard rates between the vaccinated *H(Vaccinated)* and unvaccinated *H(Unvaccinated)* groups in our case. The consistency check I applied to our case examines the simultaneous occurrence of highly disparate HRs (e.g., HR ≫ 1 and HR ≪ 1) for different chronic conditions within the same non-adjusted cohort to assess if the effect is biological or an artefact due to baseline bias.

### 2.3. Inferential statistics

After basic descriptive statistics, I list here the inferential statistics methods necessary to develop rigorous hypothesis testing procedures that add inferential confirmation (or simply rejection) to the validity hypotheses of the initial data coming from study [6]. The methods are explained succinctly, but with a listing of the corresponding null and alternative hypotheses [10],[11].

#### 2.3.1. One-Sample Z-test for schizophrenia incidence

The purpose of this inferential test is to statistically assess the validity of the Schizophrenia incidence rate reported in [6] for the vaccinated group. By comparing it against established national epidemiological benchmarks, I test if the observed deficit is within the bounds of statistical plausibility. This approach enables us to evaluate the Null Hypothesis of methodological consistency even when considering potential demographic or behavioral variations. The analysis will rely on the following key data and metrics: a) The *Observed Rate*, *P(Obs)*, extracted directly from [6], showing the 3-month Schizophrenia incidence rate in the vaccinated cohort; and b) the *Benchmark Rate*, *P(Bench)*, derived from the robust national registry study in [7], which establishes the expected annual incidence for Schizophrenia in South Korea. This annual rate is normalized to a 3-month (quarterly) period. c) the *Sample Size* (*N*), that is he exact size of the vaccinated cohort, *N(Vac)*, as reported in [6]. A summary of these figures is reported in Table 1 below.

**Table 1.** Data inputs required to compare the observed 3-month incidence proportion of Schizophrenia in the vaccinated cohort [6] against the national epidemiological benchmark [7].

**Metric**	**Value (3-month rate/proportion)**	**Source/Reference**	**Calculation of Cases**
*P(Obs)*	0.51/10,000 = 0.000051	Vaccinated Cohort [6]	1,718,999 x 0.000051 ≈ 88
*P(Bench)*	~ 2.1/10,000 = 0.00021	National Incidence Range: 2.0–2.2/10,000 (annually, normalized/4) [7]	1,718,999 x 0.00021 ≈ 361
Cohort Size (*N*)	1,718,999	Vaccinated Cohort [6]	Not applicable

In this circumstance, the test of interest aims to detect a non-plausible *deficit* in observed cases, making it a one-tailed Z-test, which can be structured as follows:

Null Hypothesis (*H0*). The observed Schizophrenia incidence proportion in the vaccinated cohort *P(Obs)* is equal to or greater than the national benchmark proportion *P(Bench)*. This assumes methodological consistency, *H0*: *P(Obs)* ≥ *P(Bench)*.

Alternative Hypothesis (*H1*). The observed 3-month schizophrenia incidence rate in the vaccinated cohort is significantly lower than the national benchmark rate, suggesting uncorrected bias, *H1: P(Obs)* < *P(Bench)*.

At this point, a correct statistical test is needed to decide *H0* vs. *H1*. This is the Z-score test statistic, which is calculated using the standard formula for comparing a sample proportion to a known population proportion:

$$Z=(P\left(Obs\right)-P\left(Bench\right))/\sqrt{P(Bench)(1-P\left(Bench\right))/N}$$ (3)

#### 2.3.2. Confidence interval calculation for bipolar disorder incidence

The objective in this case is to establish the statistical precision and plausible range of the observed 3-month Bipolar Disorder (BD) incidence rate reported in the unvaccinated control cohort of [6]. This precision will then be compared against the external 12-month national prevalence rate from [8] to test for methodological consistency. This time, the idea is to use formal statistical hypothesis testing to corroborate (or confute) the validation activity begun with simpler techniques of descriptive statistics. This analysis is carried out by comparing the 3-month observed BD incidence proportion (*P*) in the unvaccinated control cohort [6] against the 12-month Borderline Personality Disorder (BPD), yielding similar symptoms and conditions) national prevalence benchmark *P(Bench)* [8], as shown in Table 2 below.

**Table 2.** Data inputs comparing the 3-month observed BD incidence proportion (P) in the unvaccinated control cohort [6] against the 12-month BPD national prevalence benchmark P(Bench) [8].

**Metric**	**Value (Proportion or Count)**	**Source/Context**
Observed Incidence Proportion *P*	0.000139 (from 1.39/10,000)	Extracted from [6] (Unvaccinated Control Group, 3-month incidence)
Prevalence Benchmark *P(Bench)*	0.000106 (from 1.06/10,000)	Extracted from [8] (National 12-month Prevalence for similar conditions)
Control Cohort Size (*N*)	308,354	Size of the Unvaccinated Control Group reported in [6]
Observed cases (*O*)	43	Calculated from *N* x *P*

In this situation, I will use the Wald method to calculate the 95% Confidence Interval for the observed BD incidence proportion (*P*). The formula for the 95% Confidence Interval for the proportion is:

$$CI\left(95\%\right)=P\pm Z_{\alpha /2}\times SE,$$ (4)

where *P* is the observed BD incidence proportion (0.000139); *Z_α_*_/2_ = 1.96 (i.e., the critical Z-value for a 95% CI) and SE (the Standard Error) which, in turn, can be calculated as $\sqrt{\frac{\left(1-P\right)P}{N}}$.

At this point, the inferential test involves checking the relative position of the 12-month BPD prevalence benchmark *P(Bench)* within the calculated 95% CI of the 3-month BD incidence (*P*). If the benchmark is statistically consistent with the observed incidence rate, it will fall within the calculated CI.

#### 2.3.3. Two-Sample independent t-test for covariate balance (Mean age)

The purpose of this inferential test is to check a more general condition that could affect the representativeness of a given cohort. Essentially, I want to determine if the compared cohorts of [6] were statistically equivalent on a critical confounding variable, the Mean Age, prior to the intervention. Establishing this baseline balance is a necessary prerequisite for valid causal inference in non-randomized studies. In the end, following this way, it will be possible to verify if the scrutinized study yields valid or contradictory HRs. In this latter case, this would reflect the confounding effect of these pre-existing differences, rather than the biological effect of the vaccination intervention. I begin this kind of analysis utilizing the baseline statistics reported in [6] for the Mean Age of the two comparison groups reported in Table 3.

**Table 3.** Empirical baseline characteristics (Mean Age and Standard Deviation) derived from [6] used to test the assumption of covariate balance between the Vaccinated and Non-Vaccinated sub-cohorts.

**Metric**	**Value**	**Source/Context [6]**
Vaccinated Mean Age *X(Vac)*	54.67 years	Reported mean age for the vaccinated group
Vaccinated SD *SD(Vac)*	16.26	Reported standard deviation for the vaccinated group
Vaccinated Cohort Size *N(Vac)*	1,718,999	Cohort size used for the t-test
Non-Vaccinated Mean Age *X(NonVac)*	44.18 years	Reported mean age for the non-vaccinated group
Non-Vaccinated SD *SD(NonVac)*	16.28	Reported standard deviation for the non-vaccinated group
Non-Vaccinated Cohort Size *N(NonVac)*	308,354	Cohort size used for the t-test

I then use Welch's independent samples t-Test to compare the means of the two cohorts. Thus, I need to structure the following hypothesis test:

Null Hypothesis (*H0*). There is no statistically significant difference in the mean age between the Vaccinated and Unvaccinated cohorts (i.e., the groups are balanced for age): *H0*: *X(Vac) = X(NonVac)*.

Alternative Hypothesis (*H1*). There is a statistically significant difference in the mean age between the two cohorts (i.e., the groups are unbalanced): *X(Vac) ≠ X(NonVac)*.

The final t-test statistic will be calculated as:

$$t=(\overset{®}{X}(Vac)-\overset{®}{X}(NonVac))/\sqrt{\frac{SD{\left(Vac\right)}^{2}}{N(Vac)}+\frac{SD{\left(NonVac\right)}^{2}}{N(NonVac)}}.$$ (5)

We conclude this Section by reminding that the data presented here is either included directly or was extracted from the referenced documents. All calculations are easily reproducible based on the definitions provided. Further reasonable requests relative to data and calculations can be also addressed to the corresponding and sole author (email: marco.roccetti@unibo.it).

#### 2.3.4. Ethics approval of research

This study constitutes a methodological re-evaluation and secondary analysis of aggregated data published in peer-reviewed literature. Neither humans, animals, or plants are involved, and no primary individual-level data are collected or accessed for this research; therefore, institutional review board approval is not required. The national epidemiological benchmarks used for comparison are derived from public reports accessible through official South Korean health ministry repositories or through the referenced literature [7]–[9]. I commit to providing the calculation spreadsheets and statistical code used for the inferential analysis upon reasonable request.

## 3. Results

The main result of my analysis is that the application of the consistency metrics described earlier to the published data of [6] reveals three fundamental statistical paradoxes that challenge the core findings of that study. Importantly, if these statistical epidemiological checks, which highlight the three paradoxes discussed below, are performed first, any subsequent analyses or inferences would be invalid, contradictory, or, at a minimum, irrelevant.

### 3.1. Paradox I: Unexplained protective effect for schizophrenia

The researchers in [6] reported a Hazard Ratio of 0.231 for the development of Schizophrenia (ICD-10: F20-F29) in the vaccinated group compared to the unvaccinated control group. Specifically, an HR below 1.0 would suggest a protective effect, and HR of 0.231 should be interpreted as an approximately 77% reduction in the risk of developing Schizophrenia (1–0.231).

This amounts to the first paradox: There is no biological or clinical justification for a COVID-19 vaccine to confer such a profound, immediate protective effect against a chronic, neurodevelopmental disorder like Schizophrenia. To understand the mathematical source of this implausible finding, I must compare the incidence rates used to calculate this HR against known epidemiological benchmarks, like in Table 4 below.

**Table 4.** Reported and benchmark quarterly incidence rates for Schizophrenia (ICD-10: F20-F29), illustrating severe cohort selection bias.

**Condition**	**Group**	**Reported Incidence [6] (per 10,000 over 3 months)**	**Crude Ratio (Vac/UnVac)**	**National Benchmark [7] (quarterly range per 10,000)**
Schizophrenia	Unvaccinated (Control)	1.98	Not applicable	2.0–2.2
	Vaccinated (High-Risk)	0.51	0.257	2.0–2.2

It is easy to understand that the reported HR of 0.231 is extremely close to the crude ratio of the incidence rates (0.51/1.98, approx. 0.257). The small difference exists because the HR derived in [6] was from a Cox regression model, which incorporates time-to-event data and slight adjustments, whereas 0.257 is a simple rate ratio. Unfortunately, both values signify the same magnitude of disparity. Nonetheless, the strong statistical evidence of methodological inconsistency is the incidence rate observed in the Vaccinated cohort (0.51 per 10,000). In fact, this rate is: a) Nearly four times lower than the Unvaccinated Control group (1.98), and b) importantly below the stable national epidemiological benchmark (2.0–2.2), which represents the expected rate for the general population.

Given that the vaccinated group is, on average, older and less healthy at baseline, its incidence rate should logically be higher than the control group's rate, or at least comparable to the national benchmark. The observed severe deficit of new Schizophrenia cases in this cohort is the direct result of an uncorrected selection or misclassification bias at baseline. Notably, it indicates that individuals with pre-existing (prevalent) chronic Schizophrenia are systematically excluded or miscategorized from the vaccinated cohort, artificially lowering its observed incidence and mathematically forcing the resulting HR to be an implausibly low artifact.

I pass now, for a further confirmaton, to the use of the inferential statistics with the statistical tests introduced earlier in Section 2.3.1.

Starting from the definition of *Z* and substituting the real cohort data and benchmark values, I get: $Z=(0.000051-0.00021)/\sqrt{0.00021(1-0.00021)/1,718,999}\approx -14.39.$

This resulting value of the Z-Score is an extreme value that corresponds to a *p-value* ≪ 0.000001. Consequently, the Null Hypothesis (*H0*) is rejected, thus statistically validating the presence of the methodological flaw described in Paradox I.

Importantly, all the following points should be considered: 1) *Quantitative Disparity*, that is, based on the national registry data from [7], the cohort of 1,718,999 individuals should have yielded approximately 361 new cases of Schizophrenia over three months (Table 1). However, the study [6] reports an observed rate that corresponds to only 88 cases. 2) *Non-representativeness*, that is the Z-test confirms that this relevant 75% deficit (361 expected vs. 88 observed) is statistically impossible to attribute to random chance.

This inferential evidence demonstrates that the vaccinated cohort is not representative of the national population at baseline. Consequently, the resulting Hazard Ratio (HR = 0.231) reflects this initial statistical imbalance rather than a genuine biological protective effect. The methodological implications of this systematic discrepancy are further analyzed in Section 4. Rather, I recognize here two critical issues: First, possible differences of the incidence rates at the regional and national levels should be also considered. Nonetheless, while regional differences in psychiatric reporting or healthcare access can exist, the discrepancy identified here, where the observed Schizophrenia incidence is nearly four times lower than the national average, is statistically too large to be attributed to geographical or demographic variance alone. Instead, it points to a structural exclusion of prevalent cases during the initial cohort assembly. Second, I must also consider as an alternative explanation for our finding, the healthy vaccinee effect, where individuals with better baseline health or fewer severe pre-existing conditions are more likely to seek vaccination or potential differences in healthcare-seeking behavior. However, while such factors are known to influence observational data, they typically result in minor or moderate fluctuations in incidence rates. Indeed, a reduction of nearly 75% compared to the national benchmark is particularly implausible when considering that the vaccinated cohort is, on average, 10 years older and more comorbid than the control group (as statistically demonstrated later in Paradox III). In psychiatric epidemiology, a significantly older population represents a group at higher, not lower, baseline risk for chronic manifestations. Therefore, such a massive discrepancy is epidemiologically inconsistent with a purely behavioral or biological explanation. It points, instead, to a structural exclusion of individuals with pre-existing (prevalent) schizophrenia during the initial cohort assembly. Again, the resulting HR seems to reflect more this initial statistical imbalance rather than a genuine protective effect, as the new cases are likely suppressed by the systematic misclassification of the starting population.

### 3.2. Paradox II: Epidemiological inconsistency for BD

A notable inconsistency arises when comparing the reported incidence rate for BD in the control group to national BPD prevalence data, as shown in Table 5.

**Table 5.** Comparison of the reported 3-month BD incidence rate in the unvaccinated control cohort [6] against the National 12-month Prevalence Benchmark for BPD [8], illustrating the epidemiological inconsistency.

**Condition**	**Group**	**Metric**	**Reported Value [6] (per 10,000 over three months)**	**National Benchmark [8] (12-month prevalence per 10,000)**
Bipolar Disorder	Unvaccinated (Control)	3-month Incidence	1.39	Not applicable
BPD (Similar Severity)	National Population	12-month Prevalence	Not applicable	1.06

The first point to notice is that, as labeled in Table 5, a critical distinction must be made between the observation windows: The study [6] reports an incidence over a 90-day (3-month) period, while the national benchmarks refer to a 365-day (12-month) period. This temporal discrepancy makes the reported incidence of 1.39 even more anomalous, as it represents a quarterly flow of new cases that is disproportionately high compared to the annual stock of the affected population.

Ultimately, the paradox here can be explained as follows: By definition, the incidence (new cases over three months) of a severe chronic condition (BD) cannot exceed the prevalence (total existing cases) of a similar condition (BPD) over a longer period (a full year). Additionally, my choice to use the prevalence of BPD (1.06 per 10,000) [8] as a benchmark is a deliberate and conservative decision made in deference to the authors of the investigated study [6]. Since BPD is generally considered to have a higher diagnostic and prevalence threshold than BD, using it as a reference provides the most favorable margin for their findings. The reported three-month incidence of BD (1.39) in the control group of [6] is statistically implausible as it exceeds the national 12-month prevalence of a condition of similar severity, that is BPD (1.06) [8]. Nonetheless, a more direct comparison with specific BD data further validates this paradox. According to the National Mental Health Survey of Korea 2021 reported in [9], the official 12-month prevalence of BD is 0.1%, which translates to 10 cases per 10,000 population per year. When juxtaposed with this direct benchmark, the reported quarterly incidence of 1.39 per 10,000 (roughly 5.56 annually) accounts for more than 55% of the total annual national prevalence in just one quarter.

In closing this issue, the anomaly is given by a fundamental mathematical and statistical impossibility: The reported value of 1.39 (the incidence, i.e., the new cases of BD over only 3 months) is higher than the national prevalence of 1.06 (which represents the total existing cases, both old and new, over a full 12-month period for BPD), violating the epidemiological principle that quarterly incidence cannot exceed the annual prevalence of a similar disease in a stable population. This invalidates the derived HR of 0.672.

If I pass, as usually in our analysis, to inferential statistics, I can now calculate the figures announced in Section 2.3.2. In particular, the calculation of the standard Error (*SE*) yields *SE* = $\sqrt{\frac{0.000139(1-0.000139)}{308,354}}$ = 0.00002124, while the calculation of the Margin of error (*ME*) achieves *ME* = 1.96 x 0.00002124 ≈ 0.0000416.

Now, I have all the ingredients to approach the calculation of the 95% Confidence Interval limits per 10,000. In fact, the CI can be calculated as 1.39 ± 0.416 per 10,000, where its lower limit is 0.974 and its upper limit is 1.806, both per 10,000. At this point, the 95% CI for the 3-month BD incidence is straightforward: [0.974, 1.806] per 10,000. I have finally arrived where also the inferential results confirm the severity of the methodological flaw of Paradox II. In fact, I cannot dismiss the fact that I am in the presence of the following evidences: a) *Observed CI vs. Benchmark*, that is the calculated CI for the 3-month incidence rate (new cases) is [0.974, 1.806]. The 12-month Prevalence Benchmark (1.06/10,000) falls within this interval. More importantly, the direct BD 12-month prevalence benchmark (10/10,000) [9], mentioned earlier, confirms that while the study's incidence (1.39) is lower than the annual total, its magnitude (nearly 14% of the total yearly cases in only 90 days) remains epidemiologically strained, and b) *Epidemiological Inconsistency*, that is the inclusion of the 12-month BPD prevalence value inside the CI of the 3-month BD incidence rate, while statistically allowed, is epidemiologically implausible. This means that the statistical estimate for the rate of BD new cases over 90 days is so high that it is statistically compatible with the total rate of all existing BPD cases over 365 days.

In conclusion, this statistically verified anomaly supports the core argument where the observed incidence rate of 1.39 per 10,000 population of [6] is an extreme and highly improbable estimate. It points strongly to a misclassification error where a significant number of prevalent (existing) BD cases are erroneously identified and counted as incident (new) cases in the unvaccinated control cohort. This structural error consequently invalidates the derived HR (HR = 0.672).

### 3.3. Paradox III: Contradictory HRs for anxiety and more common disorders

The third and final paradox is as follows: The cohort selection bias not only creates falsely low HRs (protection, Paradox I) but simultaneously generates falsely high HRs for common disorders, such as Anxiety. The study [6] reports an HR = 1.439 for these common disorders, suggesting a detrimental effect of COVID-19 vaccination. The simultaneous presence of HR ≪ 1 (suggesting protection for chronic disorders) and HR ≫ 1 (suggesting harm for common disorders) within the same uncorrected cohort is the clear evidence that the observed effect is not biological, but a direct confirmation of the underlying uncorrected baseline disparity. The bias manifests differently across distinct pathologies based on the baseline prevalence in the two cohorts. In this case, moving to inferential statistics and hypothesis testing (as anticipated earlier in Section 2.3.3) will give the finally convincing proof. In fact, substituting the empirical mean age, standard deviations, and cohort sizes into the t-test formula, I achieve what is shown in Table 6.

**Table 6.** Inferential test (t-test) demonstrating baseline covariate imbalance.

**Calculation component**	**Formula or value**	**Result**
Numerator (Mean Difference, MD)	54.67–44.18	10.49 years
Denominator (Standard Error, SE)	$\sqrt{\frac{16.26^{2}}{1,718,999}+\frac{16.28^{2}}{308,354}}$	0.03183
Calculated T-Statistic (t-score)	*t* = 10.49/0.03183	329.58

As seen in Table 6, the calculated t-score is 329.58. Given the massive sample size, this result corresponds to an infinitesimally small p-value (≪ 0.000001). While it is acknowledged that such an extreme t-score is partly a function of the large sample size, the absolute difference of 10.49 years represents a profound demographic and clinical gap between the two groups. The Null Hypothesis *H0* is overwhelmingly rejected, confirming the methodological weaknesses underpinning the conflicting findings of [6], which amount to the following: i) A *Statistical and Clinical Imbalance*, the t-test confirms that the two cohorts are statistically and non-randomly unbalanced on age. The disparity of over 10 years on average (54.67 vs. 44.18) is not merely a statistical artifact but a clinically meaningful difference that shifts the baseline risk profile of the cohort. This disparity is strong evidence of the selection bias arising from the data-gathering process, and ii) *Conflicting HRs*, the proven imbalance explains the conflicting Hazard Ratios. In fact, since the Vaccinated group is significantly older and more comorbid (as suggested by the high mean age), it has a naturally higher baseline risk for conditions correlated with age, such as Anxiety/Stress Disorders (HR ≫ 1). Conversely, its low incidence for Schizophrenia (HR ≪ 1) is due to the non-random exclusion of prevalent cases (as confirmed by the Z-test for Paradox I).

## 4. Discussion

The analysis presented here, leveraging basic descriptive and rigorous inferential statistics (notably the Z-test for Paradox I and II, and the t-test for Paradox III) and established epidemiological reference points, consistently demonstrates that the findings of study [6] are statistically unreliable and could compromise the validity of medical inferences if they are considered without a prior statistical check. It is a plausible hypothesis that the study's core methodological weakness lies in the control cohort construction, selected via random sampling (50% of unvaccinated individuals), a suboptimal approach in retrospective administrative data studies. This random selection, in fact, fails to account for the vast, intrinsic differences between individuals who choose to be vaccinated (often older, with more comorbidities and exhibiting higher health-seeking behavior) and those who do not.

However, beyond the results regarding COVID-19 vaccination, this study should be primarily intended as a methodological cautionary tale. It highlights how the absence of preliminary consistency checks can lead to the dissemination of epidemiological artifacts, regardless of the complexity of the statistical models employed. The following further considerations are in order.

### 4.1. Amplification of bias by advanced modeling

This case raises the critical question of what would have transpired if researchers had immediately moved past the initial biased cohort construction and applied sophisticated inference tools like (ML/DL) algorithms. The answer poses a concern: ML/DL models do not correct fundamental selection bias; they simply automate and amplify it.

Consider the potential propagation of misclassification in the Schizophrenia case. As demonstrated by the statistical inconsistencies identified in previous Sections, the ML/DL model would be trained to predict the outcome based on the feature space of the highly biased cohorts. Since the feature Vaccinated is artificially correlated with a low Schizophrenia rate (due to prevalent cases being excluded from that group, Paradox I), an ML/DL model would learn this false association. When deployed in a clinical setting, such a model would falsely flag vaccinated status as a protective factor for Schizophrenia, potentially leading to incorrect clinical risk stratification.

Furthermore, I also see the risk of *over-sensitivity to noise* in the Bipolar Case. The highly complex algorithms, designed to find subtle patterns, would attempt to find a non-linear relationship explaining the anomaly identified in Section 3.2 (Paradox II). The model might latch onto an irrelevant feature, such as a zip code or a specific primary care physician, that happens to be correlated with the underlying data-entry error. This process would yield a complex, yet non-informative, explanation that adds zero predictive value but significantly increases computational cost and model opacity.

This leads to falsely prioritized features. A ML/DL model would assign significant weight to the treatment variable (vaccination) because of the strong, albeit artificial, signal it carries (HR = 0.231 for Schizophrenia, HR = 1.439 for Anxiety). The model's complex feature importance metrics would thus mislead the investigator into believing the intervention is the primary driver of the outcome, ignoring the foundational methodological flaw that created the signal in the first place. This is a classic example of the propagation of input errors into model outputs, where the complexity of the output lends undeserved credence to flawed inputs. In essence, by skipping the basic consistency check, I risk creating high-performance predictive models that are robustly and confidently predicting an artifact.

### 4.2. Necessity of covariate balancing

The observed anomalies strongly suggest a failure to properly define prevalent cases at baseline, leading to systematic misclassification. This methodological failure requires correction that ML and Big Data analyses cannot provide *post hoc*. A critical point that must be addressed is the distinction between statistical significance and clinical relevance regarding the baseline imbalances. As identified in Section 3.3, the mean age difference between the cohorts is 10.49 years (54.67 vs. 44.18). Moreover, while the massive t-score (329.58) is admittedly a product of the large sample size, the ten-year gap represents a profound clinical disparity. In psychiatric epidemiology, a decade of difference shifts the baseline risk profile for most disorders; comparing a 44-year-old cohort to a 54-year-old cohort without rigorous stratification is epidemiologically unsound, as they represent different biological and social life stages and different comorbidity burdens.

In this context, I must also address the potential role of Cox proportional hazards models, which were utilized in the original study [6] to estimate risk. While Cox models are the gold standard for adjusting for measured confounders, such as age, sex, and baseline health status, their corrective efficacy is strictly contingent upon the quality of the input data. Statistical adjustment cannot mitigate a fundamental structural bias in the cohort assembly. If the starting line of the survival analysis is biased because prevalent cases are erroneously included in the control group or excluded from the vaccinated group, the resulting HR does not reflect a biological protective or risk effect. Instead, it becomes a mathematical artifact of the initial misclassification. No amount of multivariable regression can rectify a situation where the outcome has been structurally confounded with the baseline state.

Consequently, the reliance on complex HR calculations without first establishing a foundational balance has enabled a structural methodological error to propagate. Hence, I conclude with the consideration that any valid analysis of this kind of public health data must employ a statistically robust procedure, such as *Propensity Score Matching* (PSM) [12]. PSM is designed for retrospective studies to achieve a true covariate balance, minimizing the effect of confounding factors between the groups. This case study highlights that sophisticated statistical models are no substitute for rigorous study design and proper cohort construction.

## 5. Conclusions

In this analysis, I critically evaluated a population-based health study by returning to the fundamental principles of epidemiological consistency, a necessary precursor to advanced analytical methods like Big Data and ML [1],[2]. By systematically applying descriptive and inferential statistical methods (Z-tests, Confidence Intervals, and t-tests) to the study's reported rates and baseline characteristics [6], and juxtaposing them against established national benchmarks [7]–[9], I discovered multiple, relevant statistical paradoxes. These contradictions, including a statistically implausible protective effect for a chronic mental health condition (Schizophrenia), an epidemiologically impossible incidence rate (BD), and a massive baseline covariate imbalance (proven by the t-test), conclusively establish the presence of uncontrolled selection and misclassification biases in the cohort construction. The derived HRs are therefore numerical artifacts of this deep methodological flaw, rather than reflections of a genuine biological association. Our investigation serves as a strong admonition that the inherent complexity of computational analysis, far from correcting poor data quality, will invariably amplify and obscure underlying structural errors, especially when ML and DL models are deployed on such severely compromised inputs. Consequently, the utility and reliability of any big data-driven health research are strictly conditional upon the prior successful implementation of robust study designs and preliminary inferential statistical validation to ensure the input data is fundamentally sound and epidemiologically consistent.

## Use of AI tools declaration

The author declares that he has not used artificial intelligence (AI) tools in the creation of this article.
